# Detecting and prioritizing biosynthetic gene clusters for bioactive compounds in bacteria and fungi

**DOI:** 10.1007/s00253-019-09708-z

**Published:** 2019-03-12

**Authors:** Phuong Nguyen Tran, Ming-Ren Yen, Chen-Yu Chiang, Hsiao-Ching Lin, Pao-Yang Chen

**Affiliations:** 1 Institute of Plant and Microbial Biology, Academia Sinica, No. 128, Section 2, Academia Rd, Nangang District, Taipei City, 11529 Taiwan; 2 Institute of Biological Chemistry, Academia Sinica, No. 128, Section 2, Academia Rd, Nangang District, Taipei City, 11529 Taiwan

**Keywords:** Secondary metabolites, Biosynthetic gene cluster, Duplicate gene, Self-protection, Horizontal gene transfer, Bioinformatics

## Abstract

Secondary metabolites (SM) produced by fungi and bacteria have long been of exceptional interest owing to their unique biomedical ramifications. The traditional discovery of new natural products that was mainly driven by bioactivity screening has now experienced a fresh new approach in the form of genome mining. Several bioinformatics tools have been continuously developed to detect potential biosynthetic gene clusters (BGCs) that are responsible for the production of SM. Although the principles underlying the computation of these tools have been discussed, the biological background is left underrated and ambiguous. In this review, we emphasize the biological hypotheses in BGC formation driven from the observations across genomes in bacteria and fungi, and provide a comprehensive list of updated algorithms/tools exclusively for BGC detection. Our review points to a direction that the biological hypotheses should be systematically incorporated into the BGC prediction and assist the prioritization of candidate BGC.

## Introduction

Fungi and bacteria produce a plethora of bioactive secondary metabolites (SMs), many of which play vital roles in medicine, such as antibiotics and anticancer reagents. For instance, erythromycin, azithromycin, and penicillin are beneficial antibiotics that treat several bacterial infections in lungs, middle ears, and sexually transmitted diseases (Chen et al. 2014a; Taylor et al. 2015). Vancomycin, isolated from *Amycolatopsis orientalis*, is considered a last-resort drug for Gram-positive bacterial infections and life-threatening diseases such as severe colitis caused by *Clostridium difficile*. Salinosporamide A was first isolated and characterized from *Salinispora tropica* in 2003 and acts as a potent anticancer reagent that has entered several clinical trials for various types of cancers, including melanoma, pancreatic, and lung cancer (Feling et al. 2003; Millward et al. 2012).

Recognizing the potential benefits of SMs, scientists have long sought economical and clinically useful SMs. Traditional approaches for identification of biosynthetic pathway mainly leverage bioactivity screening to first extract the bioactive compounds with desired properties and subsequently locate the responsible genes by biochemical techniques (Luo et al. 2014). It was not long until scientists noticed that SMs are usually encoded by genes that cluster together in a genetic package, which was later referred to as a biosynthetic gene cluster (BGC). A BGC consists of genes required for the synthesis of the bioactive molecule and regulatory elements, such as transcription factors and promoters. Sometimes, it also consists of transportation genes for exportation of the produced SMs and resistance genes that prevent self-destruction in the producers (Ahn and Walton 1998; Brown et al. 1996; Medema and Fischbach 2015).

Traditional biochemical characterization approaches have come to a bottleneck in the discovery pipeline, where many of SMs prove impossible to produce or extract under laboratory conditions. Furthermore, bioactivity screening greatly depends on reference information of the existing pathways, thereby limiting the capacity to unearth novel compounds with new bioactivities. This is evidenced by the fact that during 37 years between the discovery of chinolone nalidixic acid (1962) and linezolid, the first commercially available oxazolidinone antimicrobial (2000); no new structural classes of antibiotic were introduced to the market (Bax et al. 1998; Moellering 2003; Walsh and Wencewicz 2013; Weber et al. 2003). In contrast, genomic data were able to be used for the prediction of 33,351 putative BGCs (false positive rate of 5%) in 1154 prokaryotic genomes (Cimermancic et al. 2014). The striking disparity between genetic and phenotypic potentials suggests that the limit in discovering natural products lies not in nature’s capacity but in the exploration approach.

The advent of sequencing technologies, bioinformatics tools, and synthetic biology has revitalized the discovery of “orphan clusters” whose products have yet to be characterized. Over the last couple of decades, several tools have been developed for secondary metabolite gene mining (see Table 1 for list of bioinformatics tools). For example, an earlier version of genome mining used the localization of genes on the chromosomes across multiple genomes to predict gene clusters of specific pathways (Hamer et al. 2010). More advanced tools such as BAGEL, ClustScan, NP.searcher, SMURF, antiSMASH, ClusterFinder, PRISM, EvoMining, RODEO, and ARTS were designed to perform genome mining for BGCs (Alanjary et al. 2017; Blin et al. 2013, 2017; Cimermancic et al. 2014; Cruz-Morales et al. 2016; de Jong et al. 2010, 2006; Khaldi et al. 2010; Li et al. 2009; Medema et al. 2011; Skinnider et al. 2015, 2016, 2017; Starcevic et al. 2008; Tietz et al. 2017; van Heel et al. 2013; Weber et al. 2015). These tools implement algorithms to define BGC boundaries and to detect potential BGCs based on multiple indicators such as signature protein domains, distant paralogs of primary metabolic enzymes, and evolutionary hallmarks (Medema and Fischbach 2015). For functional characterization of biosynthetic key genes, two software programs, SBSPKS and NaPDoS, were developed for analyzing the 3D structure and predict their natural products (Anand et al. 2010; Ziemert et al. 2012). Predicted BGCs can then be reconstructed, cloned, and expressed by heterologous hosts using DNA assembly technologies (Chao et al. 2015; Cobb et al. 2013; Harvey et al. 2018; Tang et al. 2015a). The products are subsequently isolated and characterized with metabolomic techniques (Breitling et al. 2013; Halabalaki et al. 2014).

**Table 1:** Computational programs for secondary metabolite gene mining

Category	Software	Year/version	Features	User interface	Computation platform	Target organism(s)	Reference(s)
BGC prediction	BAGEL	2006/v1, 2010/v2, 2013/v3	Identify bacteriocins and RiPPs using HMM search with bacteriocin database	Web	Server	Bacteria	(de Jong et al. 2010, 2006; van Heel et al. 2013)
	ClustScan	2008	Identify BGCs using HMM search and predict product structure	GUI	Local PC	Bacteria	(Starcevic et al. 2008)
	NP.searcher	2009	Identify BGCs using BLAST and construct the structure of natural products	Web/command line	Server/local PC	Bacteria	(Li et al. 2009)
	SMURF	2010	Predict secondary metabolite biosynthesis gene clusters based on their genomic context and domain content using HMM search	Web	Server	Fungi	(Khaldi et al. 2010)
	antiSMASH	2011/v1, 2013/v2, 2015/v3, 2017/v4	Identify BGCs using HMMer3 to search experimentally characterized signature proteins	Web/command line	Server/local PC	Bacteria, fungi, plants	(Blin et al. 2013, 2017; Medema et al. 2011; Weber et al. 2015)
	ClusterFinder	2014	Identify BGCs using a hidden Markov model-based probabilistic algorithm	Command line	Local PC	Bacteria	(Cimermancic et al. 2014)
	PRISM	2015/PRISM,2016/RiPP-PRISM,2017/PRISM3	Identify BGCs using BLAST and HMMER and structure prediction using HMM	Web	Server	Bacteria	(Skinnider et al. 2015, 2016, 2017)
	EvoMining	2016	Identify BGCs using phylogenomic analysis	Command line	Local PC	Actinobacteria	(Cruz-Morales et al. 2016)
	RODEO	2017	Identify BGC and RiPP precursor peptide using HMM and machine learning	Web	Server	Bacteria	(Tietz et al. 2017)
	ARTS	2017	Uses three additional selection criteria, including BGC proximity, gene duplication and horizontal gene transfer, to prioritize antiSMASH-detected BGCs	Web	Server	Bacteria	(Alanjary et al. 2017)
Biosynthetic gene analysis	SBSPKS	2010	Analyze the 3D structure of PKS protein using BLAST and SCWRL; predict the order of substrate channeling between multiple ORFs in a modular PKS cluster based on docking domain interaction	Web	Server	Bacteria, fungi, plants	(Anand et al. 2010)
	NaPDoS	2012	Predict natural products of secondary metabolite genes using BLAST and domain phylogeny	Web	Server	Bacteria	(Ziemert et al. 2012)

As powerful as genome-guided methods might sound, they usually generate a large number of predictions, which may result in extensive wet laboratory work to characterize the BGCs (Lai et al. 2017; Lin et al. 2015, 2016). Therefore, prioritizing BGCs is crucial in reducing experimental procedures, cutting costs, and time. To accomplish this, additional features of potential BGCs to connect biological and pharmacological potentials must be incorporated to highlight BGCs with the most promising bioactivities. So far, only one fully automatic platform has been devised for this purpose, namely the Antibiotic Resistance Target Seeker (ARTS) (Alanjary et al. 2017). Three important hypotheses have been put forth to rationalize the computation of BGC priority in bacteria. While this model might be well applicable to bacterial genomes, a fungus-based platform has not yet been specifically developed.

In this review, we mainly focus on the biological background of BGC prioritization to complement most similar reviews in computation of identifying BGC or the resistance hypothesis only (in no context of BGC identification). We described clearly in this review that the biological background of BGC prioritization can be more complex than just the resistance genes. We also discuss to which extent these hypotheses might be useful for the computation of BGC prioritization in different genera. Not only do we provide (1) the most complete collection of the biological hypotheses associating with BGC formation and (2) the most updated list of bioinformatics tools exclusively for BGC prediction, our review points to a direction that future BGC prediction tools should be incorporated with the biological hypotheses, leading to the prioritization of candidate BGC for the generation of bioactive compounds.

Here, we summarize three hypotheses—based on the observation that some BGCs contain duplicated or resistance genes and the phenomena that some microbes can acquire resistance related genes by horizontal gene transfer; therefore, these hypotheses provide clues for prioritizing BGCs through bioinformatics analysis tools.

## The resistance hypothesis

**Fig. 1:**
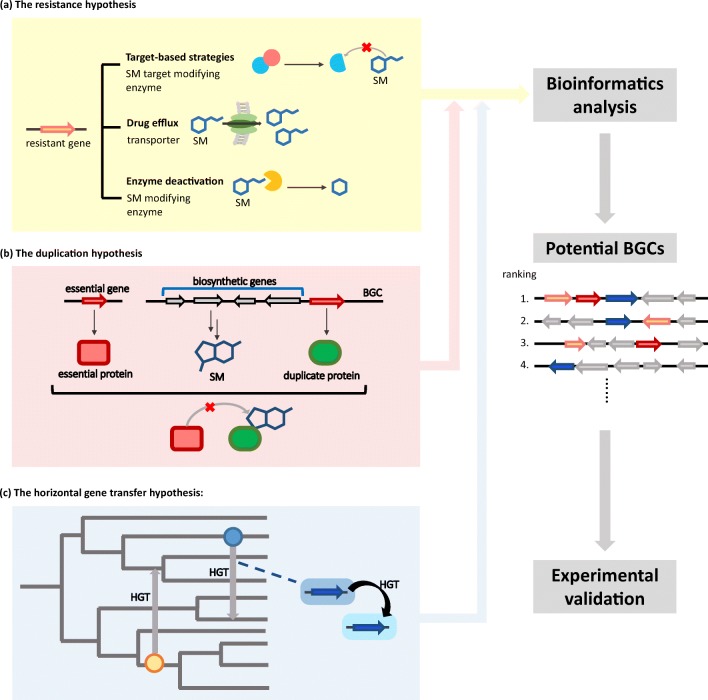
Overview of biological aspects underlying biosynthetic gene cluster (BGC) target-directed detection. Three hypotheses, numbered **a**–**c**, are presented here. **a** The resistance hypothesis comprises three notable models: target-based strategies, drug efflux, and enzyme deactivation. In the target-based strategies, the resistance gene is involved in target modification, in which the encoded protein can modify the SM-targeting protein, which is a drug receptor in drug-targeting strains or a nascent target in SM-producing strains. The resistance gene involved drug efflux encodes a transporter for pumping out the SM. For enzyme deactivation, the resistance gene encoding the enzyme modifies the SM and then deactivates it. **b** The duplication hypothesis holds that the SM producer harbors a protein isoform (duplicate protein) of an essential protein. Therefore, it protects the essential protein that the toxic SM targets by providing excess targets or proteins with greater binding affinity. **c** The horizontal gene transfer hypothesis of core genes is a potential way for microorganism to gain genetic advantage for self-protection. Bioinformatics analysis is applied to scan for BGCs that contain genes matching the three hypotheses. The output BGC candidates will be validated with experiments such as refactoring BGCs, identification of the corresponding SM product, and evaluation of biological activity

The resistance hypothesis states that within the BGC there is at least one gene conferring resistance against the potentially harmful secondary metabolites that the organism produces. The resistance mechanism can be categorized into three notable strategies, i.e., target-based strategies, drug efflux, and enzyme deactivation (Cundliffe and Demain 2010) (Fig. 1a). In the target-based strategies (e.g., target modification), the resistance gene is involved in the modification of normal drug receptors, or there is a modified version of an essential gene that is the target of the nascent SM; once transcribed, it can provide excess targets or a target with greater tolerance against the SM. As to the drug efflux, the resistance gene might encode a transporter that removes the toxic molecule from the cell or an inhibitory enzyme that intracellularly inactivates the SM.

Accumulating evidence suggests that the presence of a resistance gene acts as a self-defense mechanism for the organisms. For instance, the tylosin producer *Streptomyces fradiae* has three resistant elements, *tlrB*, *tlrC*, and *tlrD*, within the *tyl* cluster, which encodes tylosin (Cundliffe et al. 2001). The gene *tlrC,* as an example of efflux-mediated drug resistance, encodes ATP-binding protein for transporting tylosin out of cell. The *tlrB* and *tlrD* genes encode methyltransferase, a resistance determinant for methylation of 23S rRNA of the ribosomal tunnel, and thereby sterically blocks the interaction of tylosin with the tunnel wall (Vester and Long 2009), which is an example of target-based strategy. Similarly, self-immunity elements, namely homologs of *vanHAX*, are close to biosynthetic genes in *Streptomyces toyocaensis*, an actinomycete that produces the glycopeptide antibiotic A47934; *Actinoplanes teichomyceticus* producing teicoplanin (Kwun and Hong 2014; Marshall et al. 1998; Sosio et al. 2000); and vancomycin-producing *Amycolatopsis orientalis* HCCB10007 (Marshall et al. 1998; Xu et al. 2014). The *vanHAX* operon genes encode a set of enzymes that alter *C*-terminal D-Ala-D-Ala to D-Ala-D-Lac of peptidoglycan, where vancomycin and other glycopeptides bind, thereby reducing binding affinity. On the other hand, the clinical vancomycin-resistant *enterococci* encode orthologues of *vanHAX* and confer resistance (Arthur and Courvalin 1993). This modified cell wall increases the resistance to the vancomycin, which is another example of target-based strategies.

## The duplication hypothesis

As an extension of the target-based strategies in the resistant hypothesis, the duplication hypothesis claims that the resistance gene within a BGC usually shares sequence similarity with an essential gene that performs a primary function in the organism. At its core, target-based strategies and the duplication hypothesis describe very similar ideas. However, “target-based strategies” refers to a self-protective mechanism, whereas the duplication hypothesis describes one possible property of the BGCs that can be used to enhance BGC prediction.

The duplication hypothesis arises from the notion that many antibiotics’ common target sites, such as the ribosome, are also found in the producers. Hence, to protect itself, the producer harbors a copy of the target sequence with a slight modification to induce resistance against the antibiotic it produces by providing excess targets or proteins with greater binding affinity to the SM (Fig. 1b). Take *Salinispora tropica*, for example, which produces salinosporamide A to inhibit the proteasome. The proteasome, however, is also present in *S. tropica*. The gene cluster encoding salinosporamide A encloses the *SalI* gene, which shares 58% sequence identity to the proteasome *β*-subunit gene on Strop_2244. However, at the protein level, the *SalI* subunit and the typical *β*-subunit differ in only two amino acids, at positions 45 and 49. Nevertheless, when combined with the *α*-subunit, *SalI* protein forms a proteasome complex with greater binding affinity to salinosporamide A, thereby acting as an effective target modification protection against salinosporamide A (Kale et al. 2011). Recently, in a comprehensive paper published in *Nature*, Yan et al. (2018) employed the duplication hypothesis to identify the *ast* BGS encoding a dihydroxyacid dehydratase (DHAD) inhibitor in multiple fungal genomes by screening for homologues of DHAD near a BGC. The research group further expressed the BGC and confirmed the secreted natural product to be aspterric acid. It was shown that the resistance element, the *astD* gene, encodes a modified DHAD with narrower entrance to the active site, thus exerting inhibitory effects on aspterric acid.

## The horizontal gene transfer hypothesis

Horizontal gene transfer (HGT) is a widely recognized event that happens frequently among bacteria as a driving force to gain genetic advantage (Davies 1994; Ochman et al. 2000). It is postulated that at least one of the genetic elements in BGCs is horizontally acquired across species, as SM production is closely linked to ecological advantage. Natural products (NPs) such as antibiotics are often secreted as a deterrent to compete with other species sharing the same niche or to acquire nutrients from the new environment. Therefore, bacteria are bound to horizontally acquire BGCs for quick adaptation to a new environment (Fig. 1c).

The phenomenon is widely observed in many different genera, especially among *Actinobacteria*, many of which are notable secondary metabolite producers. Among 320,263 genes laterally acquired by *Streptomyces* lineages, a large proportion is genes functioning in SM and xenobiotic metabolism (McDonald and Currie 2017). This study also implied that 93% of BGCs acquired at least one gene through HGT within 50 million years, and a vast majority of BGCs were acquired from multiple sources (McDonald and Currie 2017). Similar findings were evident in *Salinispora* species, one of the genera reputed for a plethora of diverse natural compounds including products of polyketide synthase (PKS) and nonribosomal peptide synthase pathways (NRPS). A study by Ziemert et al. (2014) detected incongruence between species and gene tree in 119 out of 124 operational biosynthetic units (OBUs) that encode PKS and NRPS, indicating horizontal gene transfer at various points in 96% of biosynthetic pathways. Linear pseudochromosomes generated in this study also revealed that OBUs are assembled within genomic islands along with mobile genetic elements such as transposons that facilitate OBU exchange (Ziemert et al. 2014).

## Critical issues

### Prioritizing candidate BGCs

The concept of genome mining for BGCs is empowered by the development of many bioinformatics tools that utilize various approaches to tap into the pool of potential NPs. These tools often rely on algorithms designed to search for PKS and NRPS pathway conserved enzyme motifs (antiSMASH 1.0, SMURF, NP.searcher). However, this approach was soon demonstrated to miss out several BGCs of unknown classes. The algorithm has since been improved by many different strategies, such as looking for BGC-like patterns via data training (ClusterFinder) or a phylogenomics approach (EvoMining). Despite differences in computational approaches, all these tools result in a large number of potential BGC predictions, many of which are uncharacterized, necessitating the laborious wet laboratory work to verify the “omics” forecast. The biggest challenge is now no longer to detect BGCs but to prioritize the experimental procedures for BGCs with the most valuable biomedical potentials.

This concept of prioritizing BGCs was first introduced and validated in *Salinispora* strains by Tang et al. (2015b). In 2017, ARTS was developed and became the first fully automatic platform that exploited additional genetic features of value-added BGCs to provide a more precise prediction about the possibility of synthesizing beneficial natural products (Alanjary et al. 2017). The model employs all three aforementioned hypotheses to screen for novel drug targets. Selection criteria for potential BGCs include (i) the presence of resistance elements near a BGC, (ii) evidence of duplicate genes, and (iii) evidence of horizontal gene transfer (Alanjary et al. 2017; Freel et al. 2013; Kale et al. 2011; Thaker et al. 2014; Wright 2007; Ziemert et al. 2014). The model results in a list of BGCs with information regarding the presence of genes that match any of these three criteria. Thus, users can draw attention to the BGCs highlighted with the greatest number of hits to all screening conditions.

### Biological issues

The biological foundation of current target-directed BGC prioritization was mainly derived from observations in *Salinispora* species. While this lineage represents a large proportion of natural product producers, it certainly does not account for the diversity in nature. A number of high-value BGCs in nature do not follow the stated rules.

Regarding the resistance gene hypothesis, for instance, the *tsnR* gene responsible for resistance against thiostrepton has been identified in *Streptomyces laurentii* among ribosomal protein operons that are not closely linked to the thiostrepton-BGC (Smith et al. 1995). Besides three resistance genes colocating within the tylosin-producing cluster, the fourth element of resistance in *S. fradiae*, *tlrA* occupies an undetermined location in the genome (Cundliffe et al. 2001).

The duplicate gene hypothesis faces uncertainty in cases where different resistance mechanisms are employed. For example, in *Streptomyces kanamyceticus*, the *kanM* gene, which encodes for the AAC(6′) enzyme, lies within kanamycin-BGC. AAC(6′) can inactivate kanamycin to protect the organism from the lethal effect of kanamycin (Benveniste and Davies 1973; Kharel et al. 2004; Matsuhashi et al. 1985). In other cases, the resistance gene might code for a transmembrane transporter to export the drug or bind to the drug to sequester it from susceptible target sites (Cundliffe and Demain 2010; Le et al. 2009; Linton et al. 1994). In these examples, there is no need for the resistance gene to be a duplicate of the target sequence. Current bioinformatics tools focus on the target modification resistance mechanism since the search for duplicate genes is more computationally feasible compared to examining inactivating enzymes or transporter genes. In addition, whether transporter and enzyme-coding genes act in self-protection or biosynthesis of the secondary metabolite is elusive without experimental characterization.

Although HGT is widespread in bacterial BGCs, it is remarkable that the extent and rate of HGT remains unknown (McDonald and Currie 2017). Once thought to be the driving force of bacterial revolution, there is evidence that HGT might not be as rampant as previously believed (McDonald and Currie 2017). The acquisition of BGCs might be selectively neutral, thus presenting no genetic advantage to facilitate their possession, as evidenced by the limited spread of BGCs among only one or two strains of *Salinispora* (Jensen et al. 2007; McDonald and Currie 2017; Sieber et al. 2014). In some cases, the acquired genetic packages remain silent in the host or might not produce the intended molecules, thereby adding noise to the computational predictions from ARTS (Alanjary et al. 2017; Gogarten and Townsend 2005; Kimura 1977).

### Bioinformatics issues

Bioinformatics attempts to highlight duplicated genes greatly dependent on varying, ambiguous parameters such as cut-off points for sequence similarity and the number of duplicate genes. Sequence identity at the gene level has been reported to be as low as 58% and as high as 80% while it was observed that similarity at the amino acid level might be higher, with only 1–2 different residues (Hansen et al. 2011; Kale et al. 2011). The number of duplicates also raises certain doubts about the predictability of potential BGCs. Theoretically, a single copy of the essential gene is sufficient to protect the producers, which has also been observed in many species (Kale et al. 2011; Thiara and Cundliffe 1989). However, some genomes inherently possess two copies of essential genes via gene duplication that is associated with environmental adaptation (Bratlie et al. 2010).

In addition, current screening procedures necessitate an existing database of resistance and core genes (e.g., the Comprehensive Antibiotic Resistance Database (CARD), resistance elements) or a built-in database (e.g., core genes from the *Actinobacteria* phylum reference set that includes complete genomes from 189 species of 22 different families) (Alanjary et al. 2017). While the database is readily available for bacterial genomes, fungal genomes are less documented, which hinders the development of such BGC target-directed detection in fungi.

### Fungal genome mining

Like bacteria, fungus is another group of organisms that yields valuable bioactive compounds. Fungal genomes in general are more complicated than bacterial genomes, with more genes and BGCs. Fungal metabolic gene clusters might contain at least 15 genes and span tens of kilobases (Brown et al. 1996; Gardiner et al. 2004; Keller et al. 2005; Kennedy et al. 1999; Proctor et al. 2003). The task of prioritizing fungal BGCs hence proves more challenging and has not been developed yet.

Generally, the aforementioned hypotheses are applicable to fungi; but the extent to which each hypothesis weighs in the fungal BGC discovery pipeline is still uncertain. There is evidence for the presence of a resistance gene that is a duplicate of a target sequence in several *Penicillium* and *Aspergillus* species (Gilchrist et al. 2018; Hansen et al. 2011; Lin et al. 2013). An extra copy of inosine-5′-monophosphate dehydrogenase (IMPDH), the primary target of MPA, with 80% identity is embedded within the MPA gene cluster, while the fumagillin gene cluster possesses an additional housekeeping gene, MetAP-2, an inhibitory target of fumagillin (Hansen et al. 2011; Lin et al. 2013, 2014). Similarly, the gene cluster encoding fellutamide B, a proteasome inhibitor in *A. nidulans*, contains the *inpE* gene, whose protein shares 71% amino acid sequence similarity to a proteasome component C5. The gene cluster of aurovertins, potent inhibitors of F1 ATPase, encodes an ATP synthase which is likely to confer self-resistance (Mao et al. 2015). The presence of the *inpE* gene was later confirmed to confer resistance to fellutamide B (Yeh et al. 2016). Surprisingly, the *A. fumigatus* gliotoxin (*gli*) BGC also harbors the *gliT* gene, which encodes for gliotoxin oxidoreductase, an enzyme that converts gliotoxin into a less toxic compound (Scharf et al. 2010). *gliA* was found within the *gli* BGC to encode an efflux pump that might act in the resistance mechanism against gliotoxin (Dolan et al. 2015). The extent to which *gliT* and *gliA* contribute to *A. fumigatus* self-protection remains difficult to determine. However, there is more evidence of resistance via drug efflux than detoxifying enzyme activity at present (Keller 2015). With cases where self-protection is driven mainly by efflux or a detoxifying enzyme, the duplication hypothesis might not be applicable.

HGT is thought to be an important mode of gene transfer along with vertical transmission in fungi due to the prominent genetic instability of the fungal genome. Many studies have documented events such as translocation, deletions, inversions, and spontaneous mitotic or meiotic instability in fungi (McDonald and Martinez 1991; P. megasperma Drechs 1990; Morales et al. 1993; Pitkin et al. 2000; Sweigard et al. 1995). During genome replication for vertical transmission (sexual or asexual reproduction), these events will likely lead to the loss of essential genes. On the other hand, HGT events are independent of DNA duplication, making them a safer mode of gene transfer than vertical transmission. One mechanism fungi exploit to adapt to HGT is to cluster metabolic genes into a wholesale package that can be exchanged in a single event. There is accumulating evidence of full pathway transfers between fungi, including the sterigmatocystin gene cluster in *Podospora anserina* that was laterally acquired intact from *Aspergillus nidulans* (Slot and Rokas 2011). In addition, HGT might take place in part, such as the case of the avirulence-conferring enzyme 1 (*ACE1)* gene cluster in *Aspergillus clavatus*, where at least five genes were laterally acquired from an ancestor of *Magnaporthe grisea* (Khaldi et al. 2008). There are also some cases of interkingdom HGT, such as the ancient transfer event of 6-methylsalicylic acid-type PKS from actinobacteria to ascomycete fungi (Schmitt and Lumbsch 2009; Sieber et al. 2014).

## Concluding remarks

Traditional approaches to discover SMs are considered “top-down” methods due to their dependency on biochemical methods (Luo et al. 2014). For example, with a traditional approach, granaticin was first isolated from *Streptomyces olivaceus* in 1957 but also detected in *S. violaceoruber* based on antimicrobial testing against Gram-positive bacteria and protozoa (Barcza et al. 1966; Carbaz et al. 1957). The biosynthesis pathway that involved polyketide synthase was elucidated in 1979 by a combination of feeding experiments, chemical techniques, and it is previously described on other *Streptomyces* spp. (Snipes et al. 1979). Leveraging on this pathway, Bechthold et al. (1995) detected a 50-kb BGC in *S. violaceoruber* strain Tü22 using DNA probes derived from consensus gene sequences encoding similar catalyzing enzymes found in other actinomycetes.

The key feature of genome mining is to turn the ad hoc process of discovering SM into a high-throughput pipeline in the identification of BGC and the subsequent validations. As the number of genome sequences available will continue to rise exponentially, it is now a perfect timing for large scale genome mining. For example, the genome sequences as well as the epigenomes of black truffle was recently profiled (Martin et al. 2010; Montanini et al. 2014), together with the transcriptomes of several tissues from its developmental stages (Chen et al. 2014b), these altogether provides much more information for fungal BGC prediction and experiments that was simply too challenging in a couple decades ago. The advancement of sequencing technologies such as Pacific Biosciences and Oxford Nanopore is likely to generate genome assemblies with a lesser expense (Lasken 2012). Furthermore, the development of metagenomic analysis is also contributing to the information for microbial genome mining (Streit and Schmitz 2004).

The call for a genome-guided natural product discovery has been made since 2010, which Walsh and Fischbach (2010) referred to as version 2.0. It utilizes algorithms that are independent of known biosynthesis pathways to identify core enzymes involved in the biosynthesis of SMs via homology search algorithms such as HMMs. BGCs are then predicted by comparing nearby core genes with a set of manually curated BGC cluster rules. In addition to this model, the search for BGCs also employs the ClusterFinder algorithm, which is based on annotated PFAM domains (Cimermancic et al. 2014). This approach enables the discovery of BGCs at full capacity by taking the whole genome into account. In contrast, the conventional method omits silent BGCs that are not expressed under regular conditions and BGCs of uncharacterized compounds.

Notwithstanding that bioinformatics is an excellent tool to tackle the bottleneck problem of the traditional discovery pipeline, it often yields a myriad of BGC predictions with no ranking, making for a challenging laboratory validation procedure. ARTS is the first bioinformatics tool that incorporates three recently arising hypotheses to prioritize BGCs, including (i) the presence of resistance genes, (ii) duplicate genes, and (iii) evidence of horizontal gene transfer. It has provided selective criteria for certain species to target antibiotic-producing BGCs where target modification resistance is employed but has not been quite applicable to other species. In general, there seems to be no specific set of rules to highlight BGCs in all species: the more criteria added, the more confident the prediction is.

In the future, multiple screening criteria might be included to increase the accuracy of predictions. Another plausible approach is to base the search on function-guided rules. For example, antibiotic seekers will look for resistance elements in BGCs.
